# Anandamide Levels Fluctuate in the Bovine Oviduct during the Oestrous Cycle

**DOI:** 10.1371/journal.pone.0072521

**Published:** 2013-08-16

**Authors:** Maria Gracia Gervasi, Timothy H. Marczylo, Patricia M. Lam, Shashi Rana, Ana M. Franchi, Justin C. Konje, Silvina Perez-Martinez

**Affiliations:** 1 Laboratory de Biología de la Reproducción en Mamíferos, Centro de Estudios Farmacológicos y Botánicos (Consejo Nacional de Investigaciones Científicas y Técnicas), Universidad de Buenos Aires, Buenos Aires, Argentina; 2 Endocannabinoid Research Group, Reproductive Science Section, Department of Cancer Studies and Molecular Medicine, University of Leicester, Leicester, United Kingdom; 3 Laboratory Fisiopatología de la Preñez y el Parto, Centro de Estudios Farmacológicos y Botánicos (Consejo Nacional de Investigaciones Científicas y Técnicas), Universidad de Buenos Aires, Buenos Aires, Argentina; UAE University, Faculty of Medicine & Health Sciences, United Arab Emirates

## Abstract

Mammalian oviduct acts as a reservoir for spermatozoa and provides an environment in which they may compete for the opportunity to fertilize the oocyte. Whilst in the oviduct spermatozoa undergo capacitation essential for fertilization. Sperm-oviduct interaction is essential for sperm capacitation and is a tightly regulated process influenced by the local microenvironment. Previously we reported that the endocannabinoid anandamide (AEA) regulates sperm release from epithelial oviductal cells by promoting sperm capacitation. The aims of this work were to measure the AEA content and to characterize the main AEA metabolic pathway in the bovine oviduct and determine how these change through the oestrous cycle. In this study, the levels of AEA and two other *N*-acylethanolamines, *N*-oleoylethanolamine and *N*-palmitoylethanolamine, were measured in bovine oviduct collected during different stages of oestrous cycle by ultra high performance liquid chromatography tandem mass spectrometry. Results indicated that intracellular oviductal epithelial levels of all three *N*-acylethanolamines fluctuate during oestrous cycle. Anandamide from oviductal fluid also varied during oestrous cycle, with the highest values detected during the periovulatory period. Endocannabinoid levels from ipsilateral oviduct to ovulation were higher than those detected in the contralateral one, suggesting that levels of oviductal AEA may be regulated by ovarian hormones. The expression and localization of *N*-acylethanolamines metabolizing enzymes in bovine oviduct were also determined by RT-PCR, Western blot, and immunohistochemistry but no change was found during the oestrous cycle. Furthermore, nanomolar levels of AEA were detected in follicular fluids, suggesting that during ovulation the mature follicle may contribute to oviductal AEA levels to create an endocannabinoid gradient conducive to the regulation of sperm function for successful fertilization.

## Introduction

The oviduct plays a key role in the physiology of reproduction by providing a beneficial environment for gamete maturation and transport, fertilization and early embryo development during its subsequent passage to the uterus. It also acts as a functional sperm reservoir providing an environment where these compete for the fertilization of the oocyte.

In different mammalian species, spermatozoa are sequestered in the proximal region of the oviduct (isthmus) where they attach to epithelial cells, delaying sperm capacitation until ovulation-associated signals induce their release allowing their transit to the distal region of the oviduct (ampulla) [1].

Products of each oviductal segment (i.e. isthmus and ampulla), oviductal epithelial cells, as well as oviductal fluid are necessary for normal and efficient oviductal functions [2].

In general, mating and natural insemination in most mammalian species only take place during a specific phase of the reproductive cycle. Sperm-oviduct interactions that precede fertilization are undoubtedly influenced by the dynamics of the hormonal environment [3,4]. In addition, some molecules such as, glycosaminoglycans, present in oviductal secretions play a role in these sperm-oviduct interactions and bovine sperm capacitation [5–8]. The concentrations of these compounds in oviductal luminal fluid are under cyclic ovarian control, reaching a peak during the period of oestrus [7]. One group of such compounds is the endocannabinoids.

The endocannabinoid (EC) system refers to a group of lipid mediators, enzymes involved in their synthesis and degradation, such as *N*-acyl-phosphatidylethanolamine phospholipase D (NAPE-PLD) and fatty acid amide hydrolase (FAAH), respectively and their receptors that are involved in a variety of physiological processes including many reproductive functions. *N*-arachidonoylethanolamide or anandamide (AEA) is an EC that activates the specific cannabinoid receptors 1 (CB1) and 2 (CB2) and also activates the transient receptor potential vanilloid type I (TRPV1) ion channel. Several studies have shown that AEA is widely distributed in the mammalian reproductive tissues and fluids (oviduct, oviductal fluid, uterus, testis, seminal plasma, spermatozoa) and is involved in many reproductive functions such as sperm motility and capacitation, ovulation, embryo implantation, and oviductal embryo transport [9–15].

Other compounds that belong to the class of bioactive *N*-acylethanolamines (NAEs) include *N*-oleoylethanolamide (OEA) and *N*-palmitoylethanolamide (PEA). These are ‘endocannabinoid-like’ congeners that are thought to modulate the effects of AEA at the cannabinoid receptors by inhibiting its degradation, resulting in a prolongation of its biological effect [16–19]. Anandamide, OEA and PEA are present in human seminal plasma, mid-cycle oviductal fluid, follicular fluid, amniotic fluid and milk [20,21].

Endocannabinoids act locally, and their local concentrations are tightly regulated by a balance between their synthesis and degradation by NAPE-PLD and FAAH enzymes, respectively [22].

The EC system has recently been characterized in both mammalian oviduct and sperm cells [14,23–25]. Previously, we have demonstrated that bovine oviductal epithelial cells (BOEC) and bull spermatozoa express CB1, CB2, TRPV1 and FAAH [26,27]. Our findings also indicated that AEA signaling regulates sperm-oviduct interaction. Since sperm detachment may be due to surface remodelling by capacitation, we also demonstrated that nanomolar concentrations of AEA induce sperm capacitation in bull spermatozoa [27].

Oviductal secretions create an appropriate environment for the final maturation and capacitation of spermatozoa. The protein and lipid contents of oviductal fluid change during the oestrous cycle [4]. Recently, many studies have described an interplay between the EC system and reproductive hormones in the control of male and female reproductive physiology [28–30]. In women, AEA tone changes during the menstrual cycle; a peak of plasma AEA occurs at ovulation and positively correlates with estradiol and gonadotrophin levels, suggesting that FSH and LH may be involved in the regulation of AEA levels [31]. In addition, FAAH activity changes in mouse uterus during the estrous cycle [32], and ovarian steroids like estradiol and progesterone are negative modulators of it [33,34]. Furthermore, several studies suggest that these also exist an interplay between ECs and signals regulating pregnancy and or labour confirming a tight crosstalk between EC and sex hormones [28,35].

Of relevance is the fact that in rodents, a relationship between the spatiotemporal expression of AEA metabolic enzymes and its oviductal concentration has been demonstrated [10]. A critical balance has also been described between AEA synthesis and degradation resulting in the creation of a locally appropriate ‘anandamide tone’ required for normal embryo development, oviductal transport, implantation, and pregnancy.

We speculate that changes occur in oviductal AEA and its metabolite concentrations during the bovine oestrous cycle and furthermore that these, may be involved in sperm-oviduct interaction.

The aims of this work were therefore to firstly characterize such oviductal changes in AEA and then to investigate changes to the main AEA metabolic enzymes throughout the bovine oestrous cycle.

## Materials and Methods

### Chemicals

AEA, OEA, PEA, and deuterated internal standards of anandamide (AEA-d8), *N*-oleoylethanolamide (OEA-d2) and *N*-palmitoylethanolamide (PEA-d4) were obtained from Cayman Chemicals (Ann Arbor, MI). Acetonitrile, methanol and ammonium acetate, each of high performance liquid chromatography grade, were purchased from Fisher Scientific (Loughborough, UK) and HPLC grade water was obtained using a water purification system (Maxima ELGA, ELGA, High Wycombe, UK). All mobile phases were filtered through 0.2-µm, 4- to 7-mm-diameter PTFE filters (Waters Ltd., Elstree, UK) prior to use.

Anti-NAPE-PLD (for immunohistochemistry and western blot) and anti-FAAH (for immunohistochemistry) rabbit polyclonal antibody were obtained from Abcam and Abbiotec, respectively. The secondary goat anti-rabbit horseradish peroxidase conjugates used were from Dako (Glostrup, Denmark). An ABC detection system (ABC Elite; Vector Laboratories, Peterborough, UK) was used in conjunction with 3,3’-diaminobenzidine (Vector Laboratories) to detect the presence of immunoreactive complexes.

### Sample collection

Bovine oviducts were obtained from two slaughterhouses, Joseph Morris Butchers ltd (South Kilworth, Leicestershire, UK) and Frigorífico Rioplatense SAICIF (Buenos Aires, Argentina). Slaughterhouses gave the necessary permissions for the organ collections. The oviducts were immediately placed on ice and transported to the laboratory. According to the ovarian and corpus luteum morphology, the oviducts were classified into one of four different stages of the oestrous cycle: post-ovulatory (days 1-5), early-to-mid luteal (days 6-12), late luteal (days 13-18), and pre-ovulatory (days 19-21) phases [36]. In addition, the oviducts of each cow were separated into ipsilateral (to ovulation site/corpus luteum/dominant follicle) or contralateral. The oviducts were cleaned of surrounding tissues and the ampulla and isthmus regions were then separated and then the oviductal content was collected by squeezing (applying pressure) with tweezers. After that, BOEC were separated from the oviductal fluid by centrifugation at 800 g for 5 min at 4^o^C. The supernatants from the oviductal fluid (between 10 and 30 µl) were immediately processed for NAEs determination while the obtained cell pellets were stored at -80^°^C until further analysis.

In addition, between 100 and 500 µl of bovine follicular fluid were collected by aspiration with a syringe from follicles present in the ovaries. The follicles were classified according to their diameter into large (diameter > 1 cm) or small (diameter < 1 cm) follicles. The follicular fluid from each follicle was thereafter collected into Kimble Scintillation vials for measurement of NAEs.

### Preparation of internal standards

AEA and AEA-d_8_ were diluted in acetonitrile to make stock solutions of 5 mg/ml and 100 µg/ml, respectively. OEA and PEA were dissolved in ethanol at 10 mg/ml and 2,5 mg/ml, respectively. OEA-d_2_ (1 µg/µl) and PEA-d_4_ (1 µg/µl) stocks were supplied as ethanol stocks and stored at -20^°^C. Further dilutions were carried out in acetonitrile on ice to generate working internal standards solution of AEA-d8 (62.5pmol/ml), OEA-d2 (62.5pmol/ml) and PEA (125pmol/ml).

### NAEs determination

#### Oviductal fluid

To avoid complications arising from endogenous NAE present in the samples, extraction efficiencies from oviductal fluid were determined by the recoveries of the internal standards compared with non-extracted stocks of the same concentration. For quantification of NAEs in oviductal fluid working internal standards solution and oviductal fluid were mixed at a ratio of 1:1 (v/v) and diluted in acetonitrile to a final volume of 50 µl. The samples were mixed thoroughly and cooled at -20^°^C for 10 min to precipitate all proteins before the samples were centrifuged at 3000 g for 1 min at 4^°^C and the supernatants were transferred to HPLC sample vials ready for UHPLC-MS/MS analysis as described previously [37].

#### Epithelial cells

The BOEC from the ampulla and isthmus were resuspended in either 500 or 200 µl of lysis buffer PBS with [0.02% (w/v) sodium azide, 0.1% (w/v) SDS, 1% (v/v) Nonidet P-40, 0.5% (w/v) sodium deoxycholate], respectively and thereafter were sonicated for 30 sec. The internal standards (2.5 pmol/ml of AEA-d_8_ and OEA-d_2_; 5 pmol/ml of PEA-d_4_) were then added and the samples were diluted to 1 ml with deionized water. All samples were vortexed thoroughly and centrifuged at 15,000 g for 5 min at 4^°^C. The supernatants were collected and transferred to a clean 7-ml Kimble scintillation vial (Kinesis, St. Neots, Cambs, UK). The solid-phase method for EC extraction was performed as previously described [21]. Briefly, Oasis HLB 1cc cartridges (Waters) were preconditioned and equilibrated using 1 ml of methanol and 1 ml of water via a vacuum manifold (Waters). Samples were introduced onto the cartridges and drawn under gentle vacuum. The cartridges were washed with 1 ml of 40% aqueous methanol, and NAEs were eluted in 1 ml of acetonitrile. The eluate was dried under a constant stream of nitrogen, reconstituted in 80 µl of acetonitrile, and transferred to an HPLC sample vial ready for UHPLC-MS/MS analysis as described previously [37]. NAE concentrations were normalized to protein concentration which was determined by the Bradford method [38].

#### Follicular fluid

The follicular fluid (between 100 and 500 µl) was spiked with AEA-d_8_ (2.5 pmol/ml), OEA-d_2_ (2.5 pmol/ml) and PEA-d_4_ (5 pmol/ml) internal standards, and diluted with deionized water to a final volume of 1 ml. The samples were vigorously vortexed and the NAEs were extracted using the solid-phase method described above.

### Measurement of FAAH and NAPE-PLD mRNA expression

Total RNA was isolated from BOEC using Trizol reagent (Invitrogen) according to the manufacturer’s recommendations. The concentration of RNA was determined by spectrophotometry by measuring the absorbance at 260 nm. Only samples with a 260nm/280nm ratio greater than 1.6 were used for further analysis. cDNA was synthesized from 1 µg total mRNA using Maloney Murine Leukemia Virus Reverse Transcriptase (M-MLVRT) and random primers (Invitrogen), according to the manufacturer’s instructions in the presence of recombinant RNase inhibitor. After first-strand synthesis, PCR was performed with the following oligonucleotide primers: FAAH transcript: 5’-CTGCCAAGCAACATACCTCA-3’ (sense), 5’-GGCTGATAACCTTGGGAACA-3’ (antisense); NAPE-PLD transcript: 5’-AGATTTGGCCCTTTTGACCT-3’ (sense), 5’-CTTCACACAGTTTCGCTGGA-3’ (antisense); α-actin transcript: 5’-AGGCGGACTGTTAGCTGCGTT-3’ (sense), 5’-TGCTCGATCCAACCGACTGCT-3’ (antisense). Amplifications were performed using Taq DNA polymerase enzyme (Invitrogen).

PCR was performed as follows: 94^°^C for 5 min (initial denaturation) and for FAAH and actin 35 cycles at 94^°^C for 40 s, 55^°^C for 40 s, and 72^°^C for 40 s; for NAPE-PLD 35 cycles at 94^°^C for 40 s, 56^°^C for 40 s, and 72^°^C for 40 s. Negative controls were performed without cDNA template. PCR products were separated on a 2% (w/v) agarose gel, stained with ethidium bromide and recorded under u.v. light with a digital camera (Olympus C5060).

### Western blotting analysis

FAAH and NAPE-PLD protein expression were determined by western blotting analysis using specific antibodies. BOEC from ampulla or isthmus were incubated with lysis buffer containing 10 µg/ml leupeptin, 2 µg/ml aprotinin, 100 µg/ml soybean trypsin inhibitor, 1mM EDTA, 1 mg/ml benzamidine, 10 µg/ml dithiothreitol, 1 mg/ml caproid acid, and 1 mM sodium orthovanadate. Oviductal cells were sonicated for 30 s and then centrifuged at 2000 g for 10 min. Protein determination was assayed by the Bradford method using BSA as standard. BOEC proteins (80 µg/ml) were separated using 10% SDS-PAGE and subsequently transferred to nitrocellulose membranes. Membrane non-specific binding sites were blocked with [2% (w/v) of fat-free milk powder] and incubated with primary anti-FAAH [1:500 (v/v)], anti-NAPE-PLD [1:2500 (v/v)] or anti-actin [1:4000 (v/v)] antibodies followed by incubations with goat anti-rabbit HRP-conjugated IgG [1:10000 (v/v)]. Immunoreactive specificity was assessed by omitting the first antibody. Bands were visualized using chemiluminescence detection reagents and an Image Quant system (GE Healthcare, Buckinghamshire, UK).

### Immunohistochemistry

The procedures for immunodetection of FAAH and NAPE-PLD were performed as previously described [15]. Slides containing tissue sections were dewaxed in xylene three times for 3 min each time and rehydrated in graded alcohol followed by incubation in distilled water. Endogenous peroxidase activity was then blocked by incubation in 6% H_2_O_2_ in water for 10 min.

Oviductal sections were incubated with blocking solution (10% v/v normal goat serum) for 20 min at room temperature and incubated with anti- FAAH (1:100) or anti-NAPE-PLD (1:1000) primary antibody overnight at 4 °C. The FAAH or NAPE-PLD antibodies were incubated with normal rabbit serum (DAKO) diluted to the same concentration as control.

Samples were washed in tris-buffered saline (TBS; 0.5 M Trizma, 1.5 M NaCl, 2 mM MgCl2, pH =7.6) for 20 min and then incubated with a biotinylated secondary antibody for 30 min at room temperature. After washing in TBS, ABC Elite reagent was applied according to the manufacturer’s detailed instructions. After washing, 3,3’-diaminobenzidine was added to each sample for 5 min. Slides were then washed in distilled water for 5 min before counterstaining in Mayer’s hematoxylin for 30 s. After washing in running tap water for 5 min, slides were dehydrated in graded alcohols for 5 min each, cleared through xylene twice for 5 min each and mounted using butylphthalate xylene (DPX) mounting medium (BDH, Poole, Dorset, UK).

### Measurement of fatty acid amide hydrolase (FAAH) activity

FAAH activity was assessed as previously described [39] in BOEC from the ampulla and isthmus at different stages of the oestrous cycle. The hydrolysed [^3^H]-AA (tritiated arachidonic acid) was resolved into the organic layer of a solvent system of ethyl acetate: hexane: acetic acid: distilled water [100:50:20:100 (v/v)] mixture. The plate was exposed to iodine to identify the [^3^H]-AA areas. The distribution of radioactivity on the plate was counted in a scintillation counter by scraping off the corresponding spots detected in the plate. The area of each radioactive peak corresponding to [^3^H]-AA was calculated and expressed as a percentage of the total radioactivity of the plates. Enzyme activity is reported as nmol [^3^H]-AA/mg protein/h. Protein concentration was determined as previously described [38].

### Statistical analysis

Statistical analyses were performed using the software *Infostat 2011* (Di Rienzo J.A., Casanoves F, Balzarini M.G., González L, Tablada M, Robledo C.W. InfoStat versión 2011. Grupo InfoStat, FCA, Universidad Nacional de Córdoba, Argentina).

Data were expressed as mean ± SEM. As the data were normally distributed and presented homogeneity of variances, parametric tests were used. Comparison between groups was performed with a two-way analysis of variance (ANOVA) followed by Tukey’s honestly significant difference test. Data from oviductal fluid were assessed taking into account the site of the oviduct and the stages of the oestrous cycle. Data from epithelial cells were assessed taking into account the stage of the oestrous cycle and the region of the oviduct (ampulla or isthmus).

Spearman correlation was used to evaluate the relationship between different variables. When P<0.05 the differences were considered to be significant.

## Results

### Determination of NAEs content in the oviductal fluid during oestrous cycle

**Figure 1:**
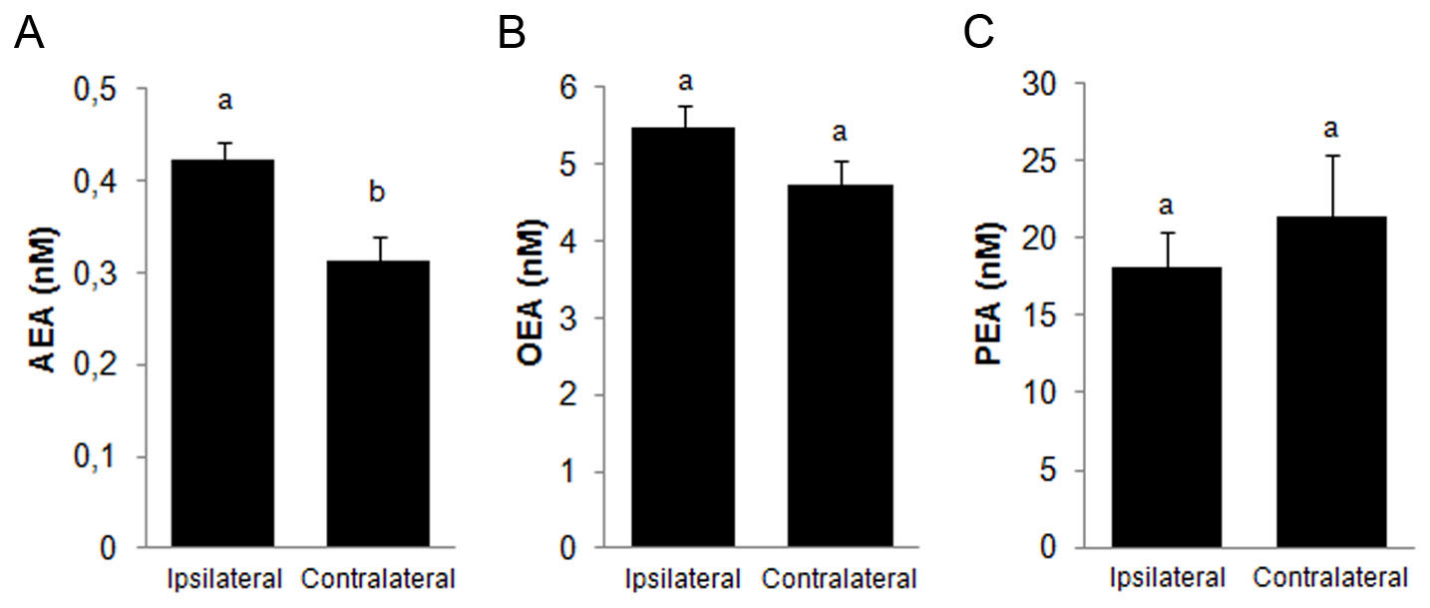
Endocannabinoid levels in the bovine oviductal fluid. Oviducts were classified as ipsilateral and contralateral oviduct to ovulation. A) AEA levels, Ipsilateral: n = 38; Contralateral: n = 34; a ≠ b, P<0.05 B) OEA levels, Ipsilateral n = 33; Contralateral: n = 30. C) PEA levels Ipsilateral n= 35; Contralateral: n= 29.

Endocannabinoid levels from oviductal fluid at different stages of the oestrous cycle were comparable (Table 1). Nanomolar AEA levels fluctuated slightly, but there was no statistically significant difference in the ipsilateral and contralateral oviducts throughout the oestrous cycle and up to ovulation. There were, however, higher AEA levels in the ipsilateral side compared to the contralateral one (Figure 1A; P < 0.05).

**Table 1:** Levels of NAEs in oviductal fluid at different stages of the oestrous cycle in the bovine oviduct.

**Days of oestrous cycle**	**Ipsilateral**				**Contralateral**			
	**AEA (nM)**	**OEA (nM)**	**PEA (nM)**	**n**	**AEA (nM)**	**OEA (nM)**	**PEA (nM)**	**n**
**1-5**	0.46 ± 0.12	6.62 ± 0.72	19.37 ± 6.32	6	0.29 ± 0.07	5.04 ± 0.65	31.53 ± 15.49	5
**6-12**	0.41 ± 0.05	4.7 ± 0.63	21.64 ± 8.19	12	0.27 ± 0.04	3.38 ± 0.41	11.74 ± 2.53	9
**13-18**	0.37 ± 0.06	5.47 ± 0.46	11.54 ± 2.05	14	0.3 ± 0.04	4.59 ± 0.34	2.41 ± 9	14
**19-21**	0.45 ± 0.13	5.09 ± 0.66	21.67 ± 7.64	6	0.39 ± 0.11	5.89 ± 0.91	19.8 ± 3.8	6

Samples of oviductal fluid were collected and NAEs were measured by UHPLC-MS/MS. Ipsilateral: oviduct ipsilateral to ovulation site/corpus luteum/dominant follicle. Contralateral: oviduct contralateral to ovulation site/corpus luteum/dominant follicle.

Oleoylethanolamide and PEA were also present at nanomolar concentrations in the oviductal fluid (Table 1). However AEA concentration was 10 and 40 times lower when compared to OEA and PEA, respectively. There were no significant differences in OEA and PEA concentrations between ipsilateral and contralateral oviducts (Figure 1B and 1C).

**Figure 2:**
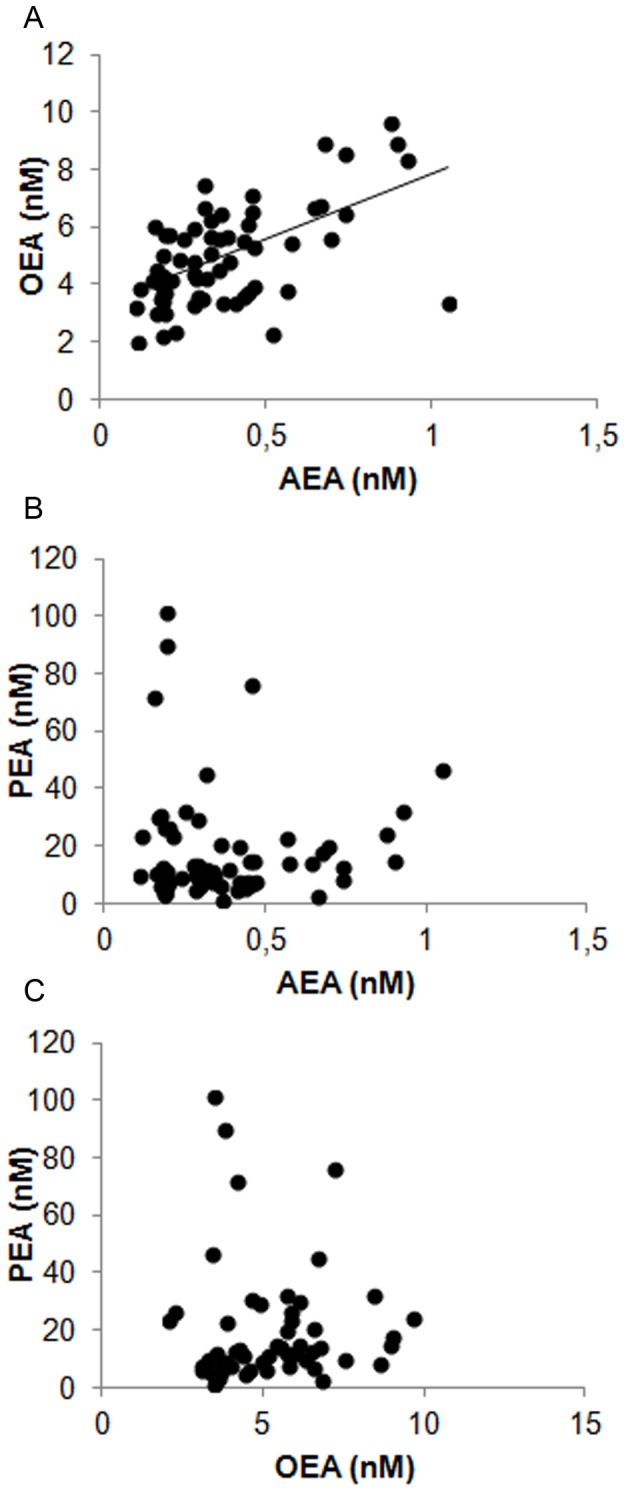
Correlation between different NAEs measured in bovine oviductal fluids. A) Positive correlation between AEA and OEA, n=63; Spearman correlation coefficient = 0.53; P<0.001. B) No correlation between AEA and PEA was observed, n= 64; P = 0.83. C) No correlation between OEA and PEA was observed, n = 55; P= 0.1.

The relationships between NAEs levels in oviductal fluid are shown in Figure 2. There were highly statistically significant positive correlations between AEA and OEA levels in oviductal fluid (Figure 2A; P < 0.001). No correlations were found between AEA and PEA or between OEA and PEA (Figure 2B and 2C).

### Measurement of intracellular NAEs content during oestrous cycle

**Figure 3:**
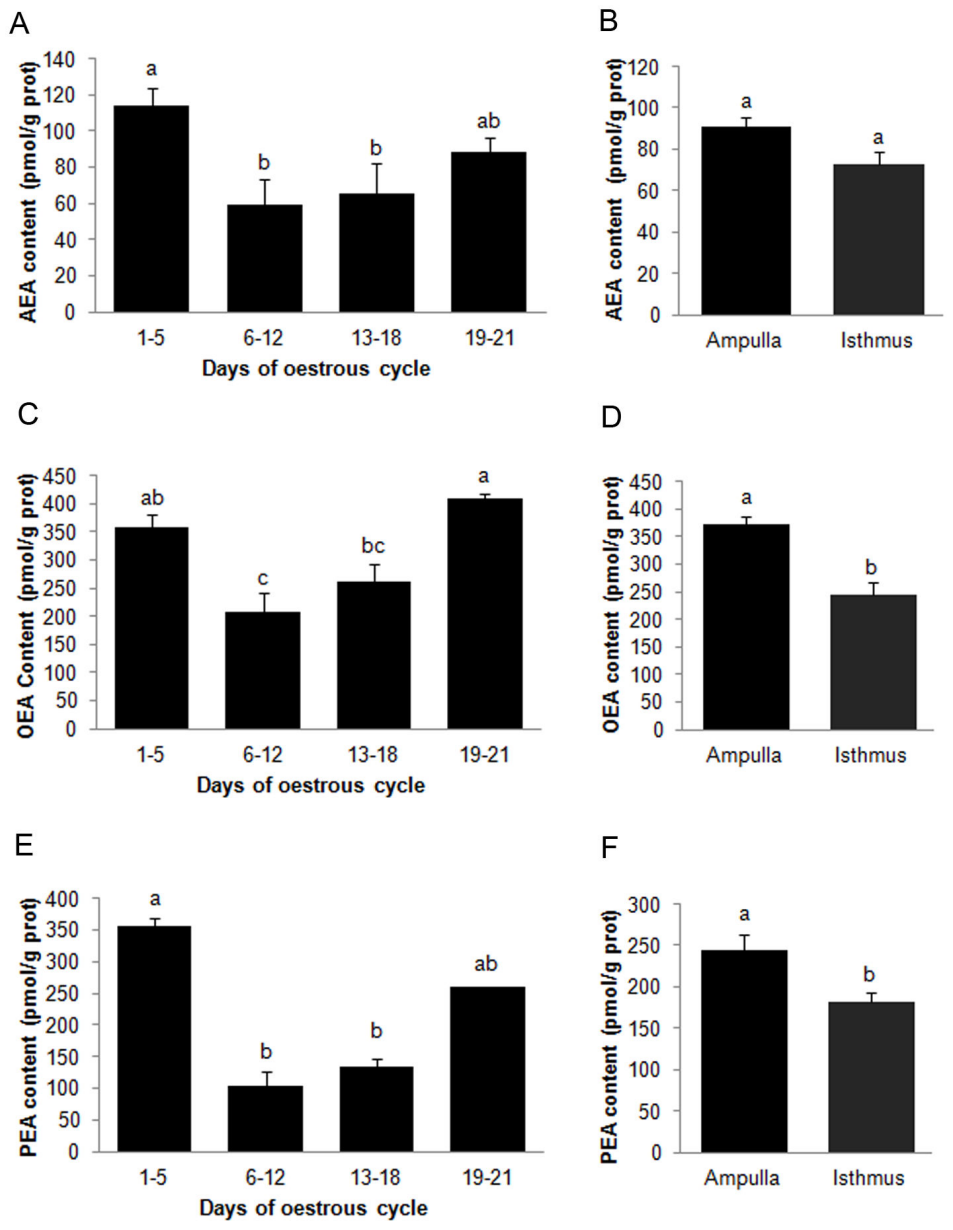
Measurement of intracellular NAEs from bovine oviductal epithelial cells. A) Intracellular AEA content in the different stages of the oestrous cycle, n = 15. B) Intracellular AEA content in different regions of the oviduct, n = 30. C) Intracellular OEA content at different stages of the oestrous cycle, n = 15. D) Intracellular OEA in different regions of the oviduct, n = 30. E) Intracellular PEA content at different stages of the estrous cycle, n = 15. F) Content of PEA in different regions of the oviduct, n = 30. a ≠ b ≠ c, P<0.05.

We also evaluated AEA, OEA and PEA content in oviductal epithelial cells from the two regions of the oviduct (ampulla or isthmus). Figure 3A shows that the highest AEA levels were found in the periovulatory stages (days 1-5 and days 19-21) compared with the early to mid-luteal and luteal stages (days 6-12 and 13-18 respectively) (P<0.05). No significant differences were found between the ampulla and isthmus (Figure 3B).

Intracellular levels of OEA and PEA demonstrated similar changes to those for AEA through the oestrous cycle, however, the levels differed significantly (P<0.05) among regions of the studied oviduct (Figures 3C, 3D, 3E and 3F). In both cases, measured levels were higher in the ampulla than the isthmus and these were elevated in the periovulatory stages (days 1-5 and days 19-21) compared to the other stages.

In contrast to results obtained for oviductal fluid, intracellular levels of AEA from BOEC did not differ significantly between the ipsilateral or contralateral side to ovulation (Table 2). However, there were significant differences in intracellular levels of PEA between the oviducts; highest PEA levels were found in the ipsilateral oviduct (Table 2).

**Table 2:** Levels of NAEs in oviductal epithelial cells from bovine oviducts.

	**Ipsilateral**	**n**	**Contralateral**	**n**
**AEA (nM)**	90.32 ± 9.26 a	31	73.21 ± 5.92 a	29
**OEA (nM)**	327.82 ± 31.36 a	31	287.06 ± 24.53 a	29
**PEA (nM)**	252.34 ± 27.02 a	29	198.33 ± 28.53 b	27

Samples of BOEC were collected and NAEs were measured by UHPLC-MS/MS. Ipsilateral: BOEC from the oviduct ipsilateral to ovulation site/corpus luteum/dominant follicle. Contralateral: BOEC from the oviduct contralateral to ovulation site/corpus luteum/dominant follicle.

**Figure 4:**
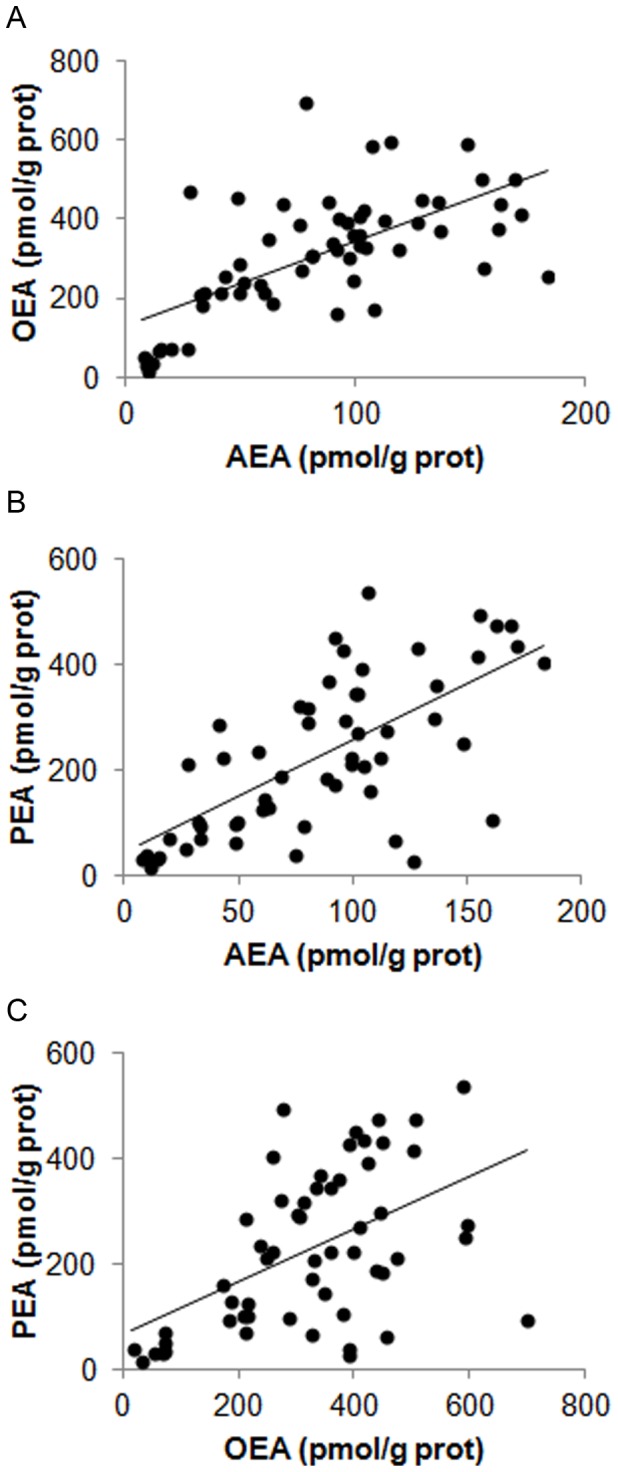
Correlation between NAE levels measured in the oviductal epithelial cells. A) Positive correlation between AEA and OEA, n = 60, Spearman correlation coefficient = 0.66; P<0.001. B) Positive correlation between AEA and PEA, n = 56, Spearman correlation coefficient = 0.67; P<0.001. C) Positive correlation between OEA and PEA, n = 56, Spearman correlation coefficient = 0.51; P<0.001.

We also determined the relationship between different NAEs in BOEC and found that AEA intracellular content correlated positively with both OEA and PEA intracellular levels (Figure 4A and 4B; P<0.001). Moreover, OEA correlated positively with PEA (Figure 4C; P < 0.001).

The results thus far indicate that AEA levels fluctuate through the different stages of the oestrous cycle in the bovine oviduct, with a higher content of AEA in the ipsilateral oviduct to ovulation.

### Measurement of AEA content from follicular fluid

At ovulation, follicular fluid is released into the peritoneal cavity and then enters the oviduct. We also determined AEA levels in the follicular fluid from different size follicles and found that AEA concentration is significantly higher in small (less than 10 mm) follicles compared to the large follicles (more than 10 mm) (Small Follicle: 1.73 ± 0.1 nM; n= 49/ Large Follicle: 0.97 ± 0.06 nM; n= 37; P<0.01).

### Characterization of NAPE-PLD and FAAH enzymes during bovine oestrus cycle

We therefore proceeded to evaluate the expression and localization of key enzymes involved in the metabolism of this EC in ipsilateral oviducts at the different stages of the oestrous cycle.

**Figure 5:**
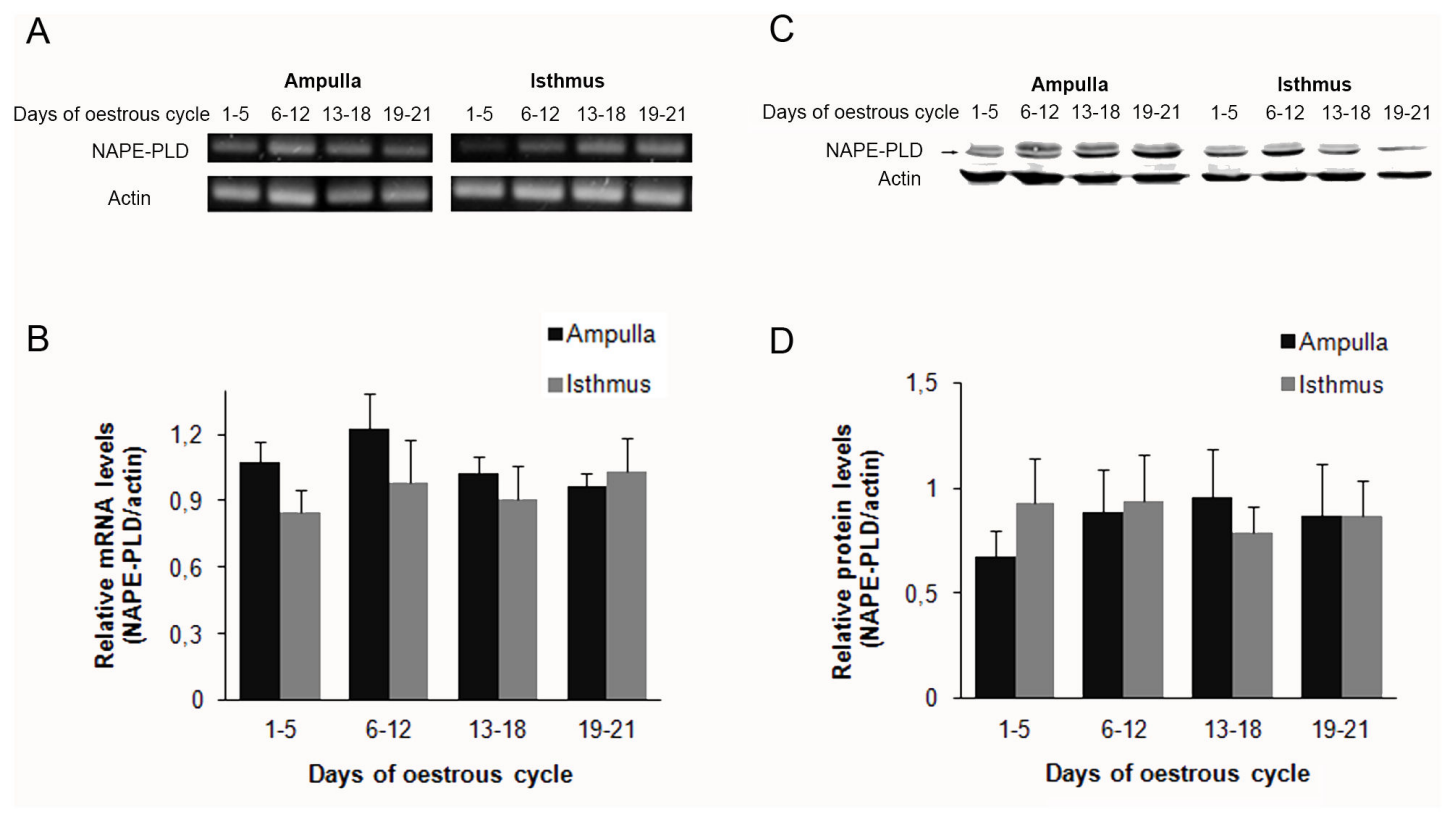
mRNA and protein expression of NAPE-PLD in the bovine oviduct during the oestrous cycle. A) Representative picture of NAPE-PLD and actin mRNA levels (n = 6). B) Relative optical densitometry (OD) analysis related to actin band. Ampulla and Isthmus: oviductal regions. C) BOEC proteins were extracted and subjected to SDS-PAGE and immunoblotted with a specific antibody against NAPE-PLD (n=6). D) Relative optical densitometry (OD) analysis was normalised to actin levels. Ampulla and Isthmus: oviductal regions.

We observed that BOEC express both the mRNA and protein of NAPE-PLD enzyme throughout the oestrous cycle. NAPE-PLD mRNA was amplified and identified by RT-PCR in BOEC from the ampulla and isthmus obtained from oviducts at different stages of the oestrous cycle as a single band at 202 bp (Figure 5A). No significant differences in the mRNA expression of the enzyme were found in the two oviductal regions through the oestrus cycle (Figure 5B). NAPE-PLD protein was detectable as a single band at the expected molecular mass of 46 KDa in all oviductal stages analyzed (Figure 5C). The protein expression of NAPE-PLD did not show any differences between the oviductal regions and the different stages of the oestrous cycle (Figure 5D).

**Figure 6:**
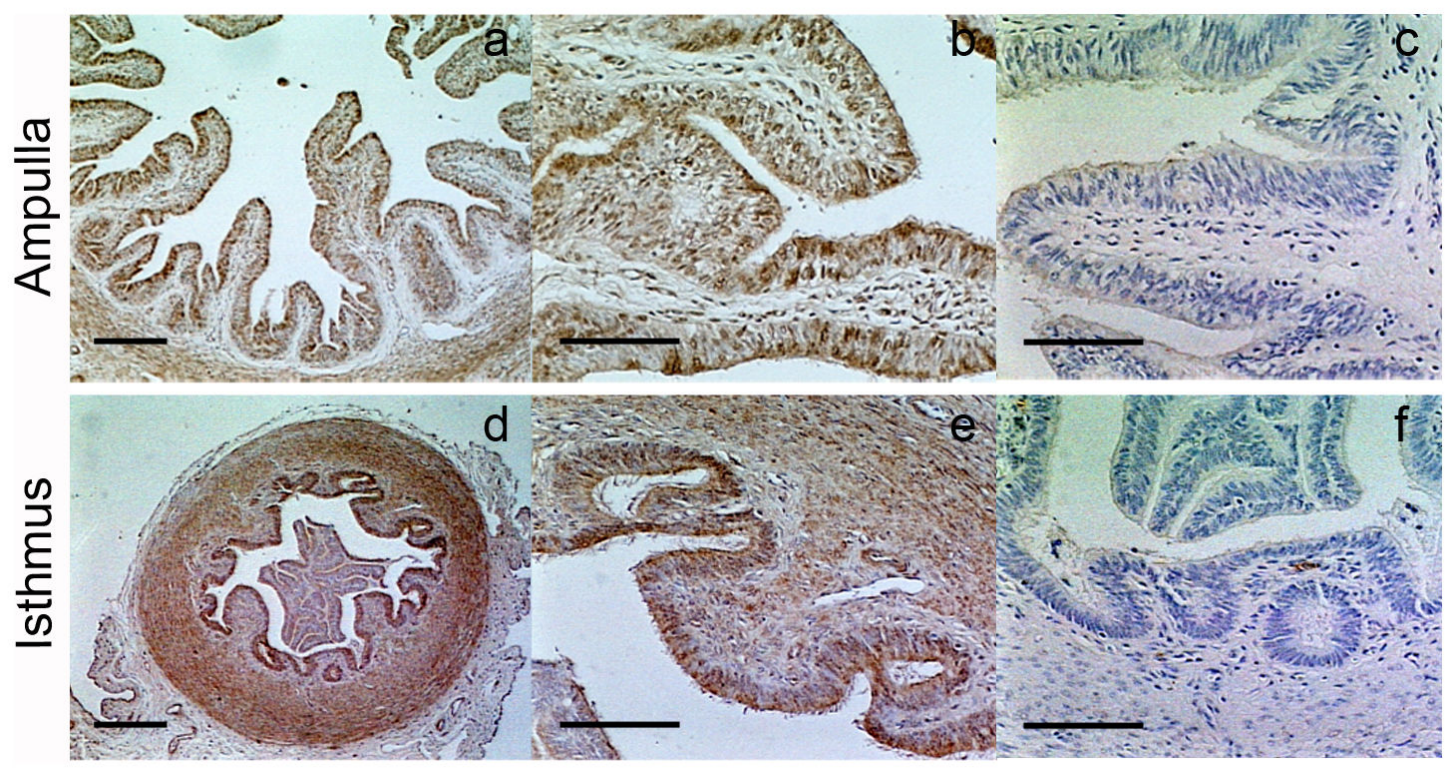
Localization of NAPE-PLD enzyme in the bovine oviduct. NAPE-PLD is mainly localized in the oviduct epithelial cells and circular muscle layer of the ampulla and isthmus. Panels c and f correspond to negative control: by the replacement of the specific primary antibody by serum from non-immunized rabbits at the same concentration. The images are representative from at least three oviducts and taken at 50x (a and d), 200x (b, c, e and f). Scale bar (a and d) = 250 µm; scale bar (b,c, e and f) = 100 µm.

Immunohistochemical studies revealed that NAPE-PLD was present in the oviduct but no differences in expression were detected between the different stages of the oestrous cycle (data not shown). In detail, NAPE-PLD was mainly localized in the epithelial and circular smooth muscle layers of the ampulla and the isthmus (Figure 6). No immunoreactivity was found when samples were incubated with the IgG fractions from non-immunized rabbits (Figure 6) or without the first antibody (data not shown).

**Figure 7:**
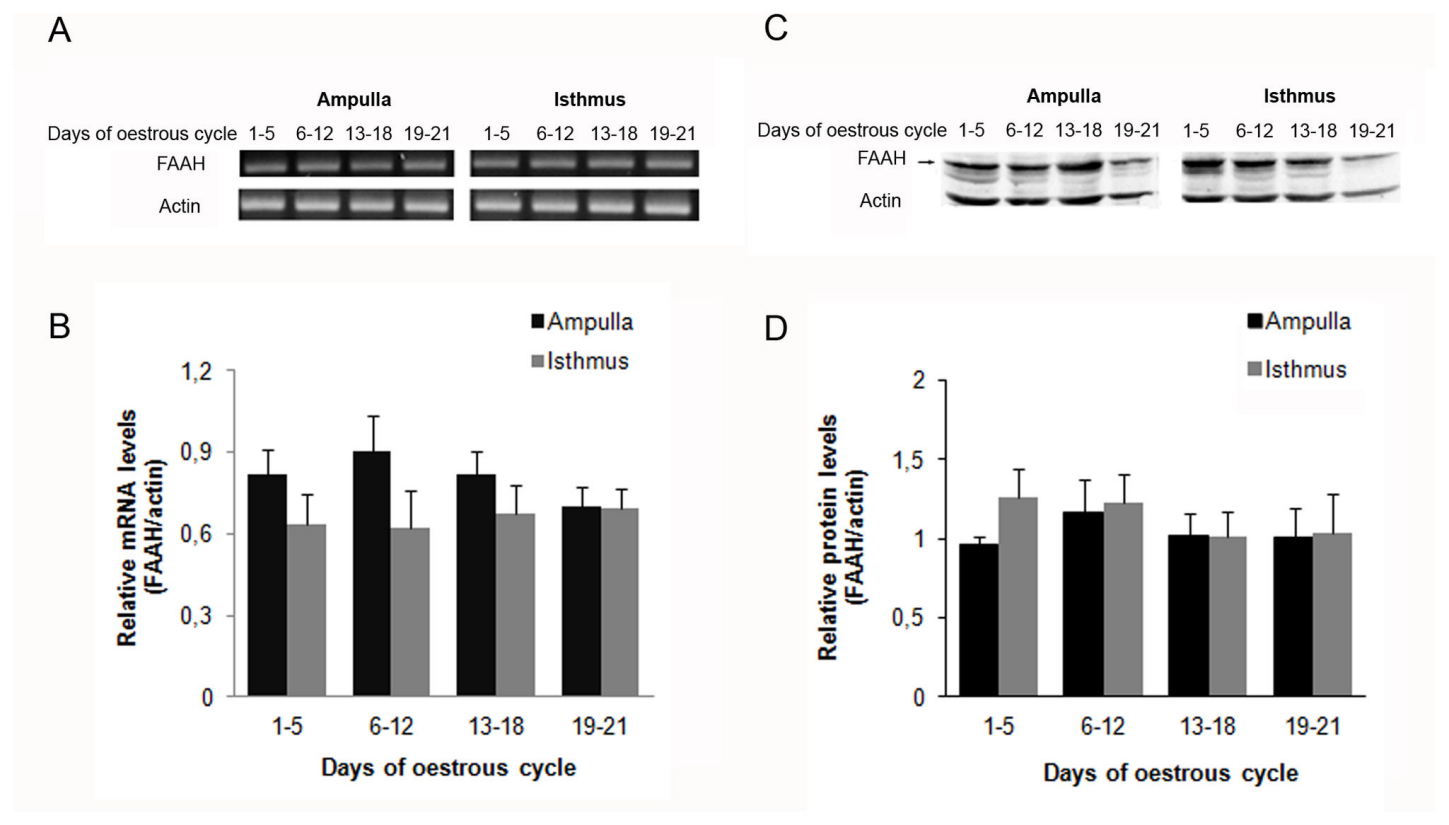
mRNA and protein expression of FAAH in the bovine oviduct during the oestrous cycle. A) Representative picture of FAAH and actin mRNAs (n = 6). B) Relative optical densitometry (OD) analysis normalised to actin expression. C) BOEC protein were extracted and subjected to SDS-PAGE and immunoblotted with a specific antibody against FAAH (n=6). D) Relative optical densitometry (OD) analysis normalised to actin expression. Ampulla and Isthmus: oviductal regions.

FAAH mRNA was also amplified and identified by RT-PCR in BOEC at different stages of the oestrous cycle as a single band at 204 bp (Figure 7A). No significant differences in the mRNA expression of the enzyme were found in the two oviductal regions and at different stages of the oestrous cycle (Figure 7B).

FAAH protein expression was readily detectable in the ampulla and isthmus at different stages and it appeared as a band at approximately 58 KDa (Figure 7C). Furthermore, when the protein expression of this enzyme was analyzed at different stages of the oestrous cycle, no significant differences were found (Figure 7D).

**Figure 8:**
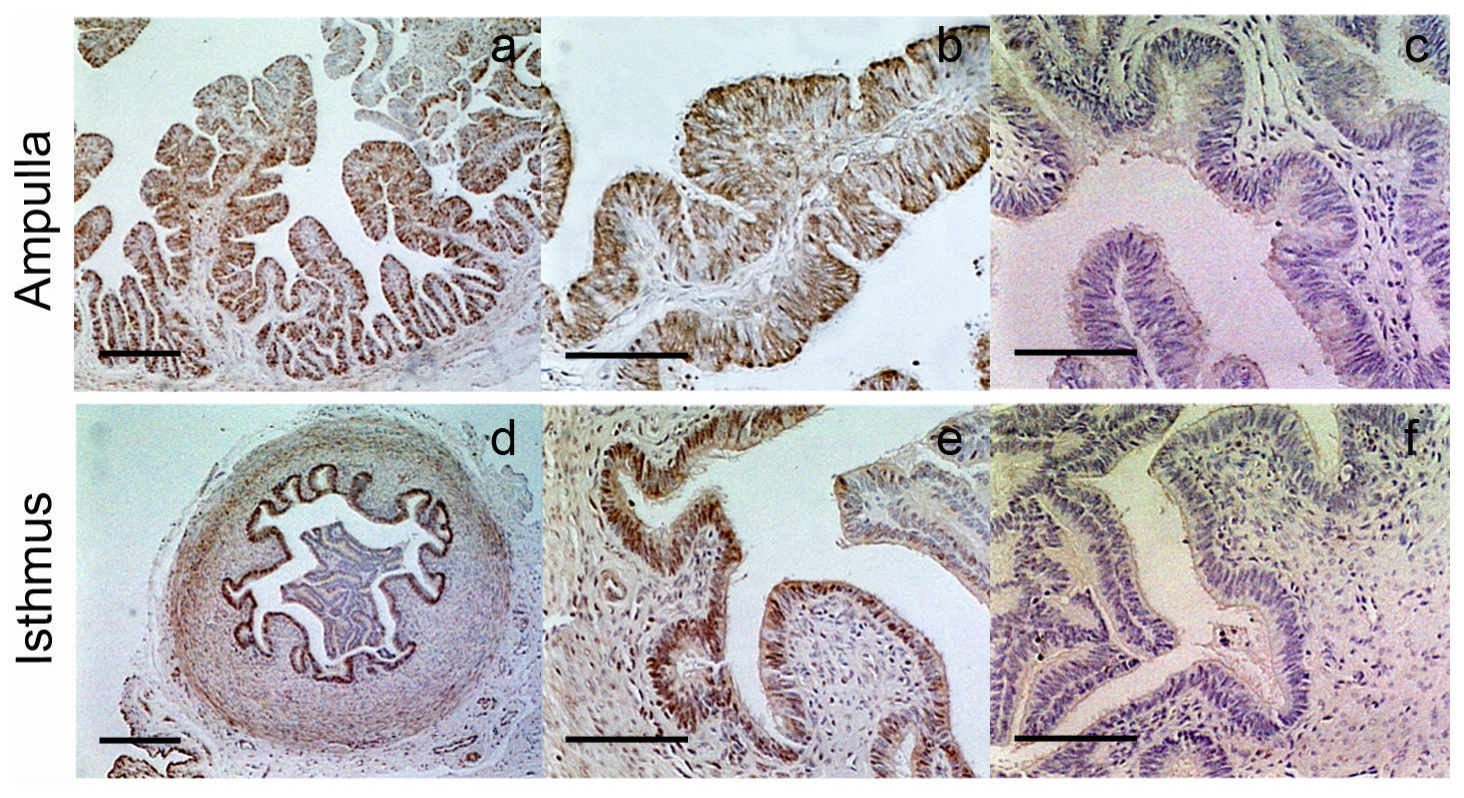
Localization FAAH enzyme in the bovine oviduct. FAAH enzyme was also localized in the epithelial and circular smooth muscle layers of the ampulla and isthmus. Panels c and f correspond to negative control: by the replacement of specific primary antibody by serum from non-immunized rabbits at the same concentration. The images are representative from at least three oviducts and taken at 50x (a and d), 200x (b, c, e and f). Scale bar (a and d) = 250 µm; scale bar (b,c, e and f) = 100 µm.

FAAH localization was also evaluated in the two regions of the oviduct during the oestrous cycle. FAAH enzyme was localized in the epithelial and circular smooth muscle layers of the ampulla and isthmus (Figure 8) with no changes between the stages of the oestrous cycle (data not shown). No immunoreactivity was found when slides were incubated with IgG fractions from non-immunized rabbits (Figure 8) or when the first antibody was omitted (data not shown).

Furthermore, FAAH activity was measured in BOEC from the two regions of the oviduct during the oestrous cycle. No statistically significant changes were found either between different oestrous cycle stages or between regions (Table 3).

**Table 3:** FAAH activity at different stages of the oestrous cycle in the bovine oviduct.

**Days of oestrous cycle**	**Ampulla**		**Isthmus**	
	**FAAH activity (nmol AA/h/mg prot)**	**n**	**FAAH activity (nmol AA/h/mg prot)**	**n**
**1-5**	112.30 ± 27.96	5	104.83 ± 16.44	5
**6-12**	83.90 ± 14.58	4	71.23 ± 7.76	3
**13-18**	91.70 ± 16.14	4	95.02 ± 22.70	5
**19-21**	89.92 ± 12.53	5	89.32 ± 16.18	5

FAAH activity was assessed in BOEC from the ampulla and isthmus at different stages of the oestrous cycle.

## Discussion

In the present work we detected, for the first time, nanomolar levels of NAEs in the bovine oviduct. We also found that AEA, OEA and PEA levels fluctuated during the oestrous cycle, suggesting that oviductal NAEs concentrations could be controlled by sex hormones.

We have previously reported that AEA is a key molecule involved in the regulation of sperm release from oviductal epithelia by promoting sperm capacitation in bovines [26,27,40]. Ovarian endocrine control is considered the overall key to completion of capacitation and sperm release from the oviduct [4], therefore we sought to establish whether oviductal AEA levels were modified during the oestrous cycle in the bovine oviduct.

Our results indicate that AEA levels from oviductal fluid were higher at the ipsilateral oviduct to ovulation compared to the contralateral oviduct. Moreover, AEA levels fluctuated during the stages of the oestrous cycle in oviductal fluid with the highest AEA content found during the periovulatory period in oviductal epithelial cells. This suggests that AEA may be synthesized locally in the oviduct under gonadal estrogenic influence delivered locally. It has been reported that hormones of low molecular weight such as steroids and prostaglandins can reach the oviduct from the ovarian vein by counter-current transfer to the oviduct branch of the ovarian artery [4]. Our results agree with those reported by [41] who found the highest plasma AEA level at the time of ovulation in women suggesting a closer association between AEA and the estradiol peak at ovulation. In human endothelial cells, Maccarrone, et al. also demonstrated that AEA release is stimulated by estradiol, through the stimulation by NAPE-PLD and inhibition by FAAH, supporting the possibility that estradiol and AEA levels may also be closely linked in these cells [42,43].

Our data indicate that OEA and PEA also fluctuate during the oestrous cycle with the highest levels found during the periovulatory phases. Interestingly, AEA levels positively correlate with those of OEA in oviductal fluid, however, the intracellular AEA content correlated both with OEA and PEA levels. This suggests that in the bovine oviduct, all three NAEs may be required for oviductal function.

Currently, the precise role that the EC-like compounds play in the bovine oviduct is unknown. Several authors consider OEA and PEA as “entourage” compounds that may enhance the receptor-mediated signaling of AEA, in which these NAEs inhibit AEA degradation through their ability to compete with AEA for FAAH, thereby leading to an enhancement of AEA effects [44–46].

Considering that NAE levels fluctuated through the different stages of the oestrous cycle in the bovine oviduct, we evaluated the expression and localization of key enzymes in the metabolism of this EC in ipsilateral oviducts. FAAH and NAPE-PLD are considered the “gatekeepers” of EC and EC-like NAE levels, and their activity and expression determine local concentrations of these ligands [47].

Previously, we demonstrated the expression of FAAH in BOEC from unclassified oviducts [26], and here we report for the first time the expression of FAAH and NAPE-PLD in BOEC during the different stages of the oestrous cycle. Moreover, in the present work we determined the localization of these proteins in the whole bovine oviduct. FAAH and NAPE-PLD were mainly detected in the oviductal epithelial cells, supporting the idea that the oviduct can produce NAEs and contribute to the oviductal AEA tone required for normal oviductal functions. A previous work reported FAAH and NAPE-PLD protein localization in the epithelial layer of the fallopian tube at different stages of the menstrual cycle [48].

Moreover, no differences in neither messenger nor protein expression of FAAH and NAPE-PLD were found when we studied the possible modulation of these enzymes during the oestrous cycle. Oviductal FAAH activity was also not modified during the oestrous cycle.

We cannot, however, rule out that the increase in NAEs observed in this work may be secondary to changes in NAPE-PLD activity. Several studies show opposing effects of ovarian hormones in the regulation of NAPE-PLD and FAAH [34,49–52] suggesting that it is hard to predict the effects of the hormones involved in the regulation of the oestrous cycle on AEA levels based upon the expression of its metabolic enzymes. Also, a number of other synthetic pathways such as a phospholipase C or a secretory phospholipase A_2_ have been proposed in neuronal tissues [53,54] and that have not been investigated in reproductive tissues which may be modulating AEA levels. Thus, we thought that direct measurement of AEA content may be more appropriate.

Although NAPE-PLD and FAAH are the main enzymes regulating NAEs levels, other enzymes, such as cyclooxygenase-2 and i-acylethanolamine-hydrolysing acid amidase have previously been reported to be involved in EC metabolism [55].

Another possibility is that AEA comes from follicular fluid released at the time of ovulation. The entry of the products of ovulation into the ampulla may catalyse the process of sperm release from the functional reservoir [4]. Our results indicate that bovine follicular fluid contains AEA at nanomolar concentration, and thus this might be a source of AEA in the oviductal fluid during the periovulatory stages. These results are in agreement with those of Schuel et al. who reported nanomolar AEA levels in oviductal and follicular fluids obtained at the mid-cycle of the human menstrual cycle and suggest that tightly regulated EC levels are crucial for events related to fertilization, with special emphasis on sperm acquisition of fertilizing ability [20].

Furthermore the presence of AEA in follicular fluids suggests that during ovulation, the mature follicle may contribute to oviductal AEA levels to create an EC gradient conducive to the regulation of sperm function. The EC gradient was previously described in the reproductive tract and seems to regulate both male and female reproductive physiology. For example, a functional 2-AG gradient regulates mouse epididymal sperm motility [56] while a gradient of AEA controls the oviductal transport of embryos and their uterine implantation in the mouse [28,57]. The fact that AEA levels measured in mature follicles were lower than those measured in primary follicles suggests a fine regulation of the AEA metabolism during folliculogenesis. El-Talatini et al. reported in humans that FAAH expression is lower in immature follicles than mature ones, suggesting that the AEA levels from follicular fluid may be regulated by this enzyme [15].

Sperm are sequentially exposed to different reproductive fluids such as oviductal and follicular fluid and secretions of the granulosa cells surrounding ovulated eggs all along the female tract up to the site of fertilization in the oviduct [58]. The presence of NAEs in these fluids and the detection of cannabinoid receptors in bovine spermatozoa [26,27] imply that NAEs-signaling may regulate sperm capacitation and fertilizing potential within the bovine reproductive tract.

Our observations suggest that oviductal NAEs levels may be controlled during the oestrous cycle in bovines and are important for further understanding of the role of EC (especially AEA) in the regulation of sperm and oviductal functions in bovines. Further studies investigating the role of molecules, such as estradiol, in the regulation of AEA levels are needed to understand the fine regulation of the EC system in the bovine oviduct.
